# No Evidence of Association Between Anti–Tumor Necrosis Factor Treatment and Mortality in Patients With Rheumatoid Arthritis: Results From the British Society for Rheumatology Biologics Register

**DOI:** 10.1002/art.27660

**Published:** 2010-11

**Authors:** Mark Lunt, Kath D Watson, William G Dixon, Deborah P M Symmons, Kimme L Hyrich, British Society for Rheumatology Biologics Register Control Centre Consortium, British Society for Rheumatology Biologics Register

**Affiliations:** Manchester Academic Health Sciences Centre, University of ManchesterManchester, UK

## Abstract

**Objective:**

To study the association between anti–tumor necrosis factor (anti-TNF) therapy and mortality in a national cohort of patients with rheumatoid arthritis.

**Methods:**

We prospectively followed up 12,672 patients who were beginning anti-TNF therapy and 3,522 biologic-naive patients receiving disease-modifying antirheumatic drugs (DMARDs) until either July 31, 2008, or death, whichever occurred first. Notification of death and cause of death was received from the UK National Death Register. Mortality was compared using Cox proportional hazards models. Inverse probability of treatment weighting was used to adjust for the confounding effects of baseline differences between groups, including age, sex, disease severity, disability, and comorbidity. Missing baseline data were accounted for using multiple imputation.

**Results:**

When compared with the DMARD cohort, the anti-TNF cohort was younger (median age 57 years versus 61 years), had greater disease activity (median Disease Activity Score in 28 joints 6.6 versus 5.1), and had greater disability (median Health Assessment Questionnaire score 2.1 versus 1.6). Patients in the DMARD cohort were more likely to have a history of myocardial infarction (4.8% versus 3.1%) and chronic obstructive pulmonary disease (8.1% versus 4.8%) but were less likely to have had depression (16.5% versus 18.9%). There were 9,445 and 50,803 person-years of followup in the DMARD and anti-TNF cohorts, respectively, during which time 204 DMARD-treated and 856 anti-TNF–treated patients died. The weighted mortality hazard ratios in the anti-TNF cohort were as follows: all-cause 0.86 (95% confidence interval [95% CI] 0.64–1.16), circulatory disease 0.73 (95% CI 0.44–1.23), neoplasm 0.65 (95% CI 0.39–1.09), and respiratory disease 0.81 (95% CI 0.36–1.83).

**Conclusion:**

Our results indicate that, compared with standard DMARD therapy, treatment with anti-TNF therapies was not associated with an increase in mortality.

Rheumatoid arthritis (RA) is a chronic systemic inflammatory condition affecting the joints and other connective tissues. In addition to chronic disability, RA is associated with increased mortality (1). The leading cause of excess mortality is cardiovascular disease, and other common causes include infection, respiratory disease, and some malignancies. The reasons behind this increased mortality are likely to be multifactorial and may include the effects of chronic inflammation, disability, and comorbidity. The effects of concurrent immunosuppressive therapy also cannot be ruled out, although the results of previous studies have suggested that the control of inflammation with methotrexate (MTX) may improve mortality (2,3).

Recently a new therapeutic approach to RA has been introduced. Unlike previous immunosuppressive agents, which offered a blanket approach to immunosuppression, these new targeted therapies, including anti–tumor necrosis factor (anti-TNF) agents, are directed at single components of the immune response. In the 10 years since their license, these drugs have been shown to significantly improve the signs and symptoms of RA and can improve disability (4–9). Whether or not these treatments can also improve the mortality rates in RA remains largely unknown. The findings of recent studies have suggested that these drugs may be associated with an increased risk of serious infection, particularly in the first few months of therapy (10), which may therefore increase the risk of death when compared with standard nonbiologic disease-modifying antirheumatic drugs (DMARDs). However, data from observational studies have also demonstrated that anti-TNF therapy may decrease the risk of new cardiovascular events (11,12), particularly among those patients who experience improvements in their disease activity, thus potentially reducing overall mortality.

Few studies have investigated the risk of all-cause mortality among patients receiving anti-TNF agents. Both a Swedish study (13) and a Spanish study (14) showed a significant reduction in mortality among patients treated with anti-TNF compared with patients receiving nonbiologic DMARDs. However, one of the challenges in assessing mortality risk within an observational study is taking into account those factors that may be associated with both the prescription of the anti-TNF therapy and the risk of death (confounding by indication). Patients who receive anti-TNF therapies often, by definition, have the most severe disease, characterized by high levels of disability, which is also a significant risk factor for premature death (15). Conversely, patients with high levels of baseline comorbidity (who are therefore at high risk of side effects) are preferentially not prescribed anti-TNF therapy (confounding by contraindication) but rather may remain on standard DMARD therapies alone. This may result in anti-TNF death rates significantly lower than those among non–anti-TNF–treated patients. To explore this further, we compared all-cause mortality between a cohort of patients starting anti-TNF therapy and a cohort of patients with active disease receiving standard DMARD therapy, using inverse probability of treatment weighting (IPTW) to allow for differences in baseline risk of death.

## PATIENTS AND METHODS

### Patients

Patients included in this study were participants in the British Society for Rheumatology Biologics Register (BSRBR) (16). Established in 2001, the BSRBR has been actively recruiting patients with physician-diagnosed RA who are starting 1 of the 3 available anti-TNF therapies: etanercept, infliximab, or adalimumab. In the UK, the use of anti-TNF agents is restricted to those patients with active disease, defined as a patient with a Disease Activity Score in 28 joints (DAS28) >5.1 (17), despite treatment with at least 2 standard DMARDs, 1 of which should be MTX (18). The primary aim of the BSRBR is to assess the long-term safety of these agents in patients with RA, with a goal recruitment of at least 4,000 patients starting each of the 3 therapies. This target number of patients was reached for etanercept in May 2005, for infliximab in 2007, and for adalimumab at the end of 2008. Up until 2005, when patients receiving all 3 anti-TNF therapies were being actively recruited for the study, it was estimated that ∼7% of all RA patients in the UK were receiving anti-TNF therapies (19) and that the register was recruiting ∼80% of that total.

In addition to patients treated with anti-TNF therapies, the BSRBR is recruiting and following a cohort of biologic-naive patients with RA who have active disease (DAS28 >4.2) and who are receiving standard nonbiologic DMARD therapy, again with a goal of 4,000 patients. Patients in this cohort were recruited from 29 rheumatology centers across the UK (see Appendix A). Recruitment of this cohort was completed in March 2009, and subjects continued to be followed up, even if they stopped or changed DMARD therapy. The one exception was subjects in the comparison cohort who subsequently started anti-TNF therapy. These patients were followed up in the comparison cohort until the date they were switched to a biologic agent and were followed up in the biologic cohort from the date of the first dose of anti-TNF. If a patient in the DMARD cohort was switched to a non–anti-TNF biologic agent as their first biologic agent, they were censored at the time of switch and were not followed up further. Patients could not switch from the anti-TNF cohort to the DMARD cohort.

### Assessments

All patients, both anti-TNF and comparison patients, were followed up in a similar manner. At the start of anti-TNF therapy (or at the time of registration into the DMARD cohort), a baseline questionnaire was completed by the medical team that detailed diagnosis, disease activity, past and current antirheumatic therapies, and other comorbidities. Comorbidities were selected from a list that included hypertension, angina, myocardial infarction, stroke, epilepsy, asthma, chronic obstructive pulmonary disease (COPD), peptic ulcer disease, liver disease, renal disease, diabetes, depression, and previous neoplasm. The severity of each condition was not specifically recorded. Only comorbidities recorded at baseline were included in the analysis. Patients also completed a Health Assessment Questionnaire (HAQ) adapted for British use (20).

Followup took place at 6-month intervals for the first 3 years and annually thereafter. At each followup visit, the hospital staff completed a questionnaire detailing any changes to antirheumatic therapy, current disease activity, and any new adverse events, including death. For the first 3 years of the study, the patient also completed a diary every 6 months, detailing any hospital admissions, as well as HAQ scores. Finally, all patients were flagged by the National Health Service (NHS) Central Register, part of the NHS Information Centre for Health and Social Care (NHS-IC) (online at http://www.ic.nhs.uk/). In the UK, there is a statutory requirement to report all births, deaths, and cancers to the Central Register. In cases of death, this must also include the underlying and contributing causes of death. These are all coded centrally according to the International Statistical Classification of Diseases and Related Health Problems, Tenth Revision (ICD-10). The Central Register database collects from multiple possible sources. In the event of a death, the coroner, hospital, or general practitioner submits information, including cause of death, to the local health authority, which electronically passes this information on to the Central Register on a weekly basis. Researchers can “flag” their subjects with the NHS-IC, which will then provide, on a quarterly basis, the details of all deaths, including a copy of the death certificate. Even if a patient is lost to followup, the details of that patient's death will be provided to researchers by the NHS-IC.

The study received ethical approval from the North West Multi-Centre Research Ethics Committee (MREC 00/8/53), and all patients provided written informed consent.

### Statistical analysis

The primary outcome measure in this study was death. Mortality rates among the anti-TNF–treated and the comparison cohorts were compared using Cox proportional hazards models and are presented as hazard ratios (HRs) and 95% confidence intervals (95% CIs). Patients were followed up from the date they first received an anti-TNF drug (or the date of registration for the comparison cohort) until either July 31, 2008, or death, whichever came first. Patients receiving anti-TNF therapy were followed up even if they discontinued the drug or switched to an alternative biologic agent. All-cause mortality and cause-specific mortality were analyzed for neoplasm (ICD-10, Chapter II), circulatory disease (ICD-10, Chapter IX), and respiratory disease (ICD-10, Chapter X). In all cases, the underlying cause of death, obtained from the death certificate, was used in the analysis. All analysis was performed using Stata 10.1 (StataCorp).

#### Adjusting for baseline differences between treated and untreated patients

Since this was an observational study, patients were not randomized to their treatment groups, and so, the anti-TNF–treated group and the comparison group may differ in variables that influence mortality, such as disease severity and comorbidity. Hence, a direct comparison of mortality rates will not reflect the effect of anti-TNF treatment. To allow for baseline differences in covariates, IPTW was used (21). Briefly, this method uses logistic regression to calculate, for each individual, the probability (*P*[x]) (also known as the propensity score) that they would be in the anti-TNF cohort given their baseline variables x. By giving a weight of 1/*P*(x) to all subjects in the anti-TNF cohort, and 1/(1–*P*[x]) to subjects in the comparison cohort, the distribution of the baseline variables in each cohort is made equal to the distribution in both cohorts combined.

To assess the need for balancing, and the extent to which it was achieved by weighting, we used a method that we have described previously (22). The degree to which an individual variable confounds the association between outcome and exposure can be assessed by measuring the unadjusted effect of treatment, then repeating the analysis, adjusting for the variable of interest to the extent possible. If the estimate of the treatment effect changes, this change implies that the covariate was a confounder. The change in the treatment effect estimate, after confounding has been removed by reweighting, gives a measure of how successful the reweighting has been. When adjusting for potential confounders, continuous variables were fitted after categorizing into quintiles, to avoid assuming a linear association between covariate and outcome.

#### Trimming

One potential problem with IPTW is that unusual subjects can be given very high weights, i.e., subjects who were very good candidates for treatment but did not receive it will have a low probability of not receiving treatment, and hence, a high weight in the analysis. This can lead to bias, if there was a reason that these subjects did not receive treatment that was not captured in the propensity score (unmeasured confounding). We therefore compared the IPTW estimate with the estimates generated by setting all weights >20 to be equal to 20, as recommended by Cole and Hernan (23), and by excluding all subjects with weights >20.

#### Multiple imputation

One problem with adjusting for a large number of variables is that it increases the probability that a given subject will have missing data on at least one variable, and hence, will be excluded from a naive analysis, which may introduce bias. To avoid this bias, multiple imputation was used, with 20 data sets being imputed using chained regression, utilizing the Ice package in Stata (24,25). The effect of the imputation was assessed by looking at the fraction of missing information, which measures how the uncertainty about an estimate has been increased by the missing data. Technically, it is given as

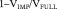

where V_IMP_ is the variance of the estimate in the multiply imputed data and V_FULL_ is the expected value of the variance of the estimate if there had been no missing data.

## RESULTS

Through July 31, 2008, 12,672 patients with RA were recruited for the cohort receiving anti-TNF treatment (4,420 patients receiving etanercept, 4,161 patients receiving infliximab, and 4,091 patients receiving adalimumab). The median age was 57 years, and 76% were women (Table 1). Patients had a high level of baseline disease activity (median DAS28 6.6), as well as high baseline levels of disability (median HAQ score 2.1). More than 50% of patients had a comorbid condition at baseline, with the most common conditions being hypertension, depression, asthma/COPD, and peptic ulcer disease.

**Table 1:** Distribution of covariates and the impact of adjustment on the estimated relative rate of mortality in patients receiving anti-TNF therapy versus patients receiving nonbiologic DMARD therapy*

	Anti-TNF–treated patients (n = 12,672)	Nonbiologic DMARD–treated patients (n = 3,522)	Change in rate ratio, %†	
			
			Unweighted	Weighted
Age, median (IQR) years	57 (48–65)	61 (52–69)	40	3
Disease duration, median (IQR) years	11 (6–19)	6 (1–15)	−6	−1
Disease Activity Score, median (IQR)	6.6 (5.9–7.3)	5.1 (4.3–6.0)	−18	2
Health Assessment Questionnaire, median (IQR) score	2.1 (1.8–2.5)	1.6 (0.9–2.1)	−18	5
Blood pressure, median (IQR) mm Hg				
Systolic	134 (120–149)	136 (122–150)	3	5
Diastolic	80 (71–87)	80 (72–87)	2	5
Sex, female	76.2	72.3	2	−1
Number of DMARDs taken previously				
1	1.8	29.7	−15	−1
2	18.5	28.5		
3	26.1	20.5		
4	20.7	11.6		
5	15	5.4		
≥6	17.9	4.3		
BMI, kg/m^2^				
<20	8.7	6.3	0	2
20–25	34.1	32.8		
25–30	32.6	35.2		
>30	24.6	25.7		
Smoking status				
Never	40.3	36.6	3	−1
Former	37.8	39.7		
Current	21.9	23.7		
History of other conditions				
Hypertension	29.7	31.8	1	−2
Angina	4.5	7.8	5	−2
Myocardial infarction	3.1	4.8	3	0
Stroke	2.1	3.2	1	0
Epilepsy	1.1	1.1	0	0
Asthma	9.9	13.5	1	−1
COPD	4.8	8.1	5	1
Peptic ulcer disease	8.5	7.3	−1	0
Liver disease	2	2	0	−1
Renal disease	2.5	2.9	0	−1
Diabetes	5.7	6.7	1	0
Depression	18.9	16.5	0	−1
Neoplasm	3.2	6.8	2	−1

* Except where indicated otherwise, values are the percent of patients. Anti-TNF = anti–tumor necrosis factor; DMARD = disease-modifying antirheumatic drug; IQR = interquartile range; BMI = body mass index; COPD = chronic obstructive pulmonary disease.

† Calculated according to the formula RR_A_ – RR_C_/RR_C_, where RR_C_ is the crude rate ratio and RR_A_ is the rate ratio after adjustment for the variable in question. This allows measurement of the extent to which the variable is confounding the rate ratio.

When compared with the anti-TNF cohort, the nonbiologic DMARD cohort (n = 3,522) was slightly older and had a higher proportion of men. Although these patients had slightly lower levels of disease activity than patients receiving anti-TNF therapy, their disease activity was still high (median DAS28 5.1). Similarly, they had moderate levels of disability (median HAQ score 1.6). A similar proportion of patients as that observed in the anti-TNF cohort had a comorbid condition, although angina, asthma, COPD, and/or previous neoplasm were more common in the nonbiologic DMARD group (Table 1). In the nonbiologic DMARD cohort, 173 patients subsequently started anti-TNF therapy during followup and were followed up for a time in both cohorts.

As of July 31, 2008, there were a total of 50,803 person-years in the anti-TNF cohort (mean followup 4 years) and 9,445 person years in the nonbiologic DMARD cohort (mean followup 2.7 years). During this time, there were 856 deaths in the anti-TNF cohort and 204 deaths in the comparison cohort. Data on the underlying causes of death, classified according to the ICD-10, were available for 98.4% of the cohort and are listed in Table 2. The leading causes of death were circulatory disease, neoplasm, respiratory disease, and musculoskeletal disease, primarily severe RA. Proportionately more people in the DMARD comparison group than in the anti-TNF group died of a neoplasm (32% versus 20%). There was a higher proportion of deaths in the anti-TNF group than in the comparison group resulting from diseases of the musculoskeletal system (15% versus 5%), primarily RA. Of note, RA was listed on the death certificate of only 31% of patients. Other causes of death were equally distributed between the 2 groups.

**2:** Underlying causes of death, as recorded on the death certificate, among patients in the anti-TNF and nonbiologic DMARD cohorts, classified according to ICD-10 chapter number*

Chapter	Blocks	Chapter title	Deaths in the anti-TNF cohort (n = 856)	Deaths in the nonbiologic DMARD cohort (n = 204)
I	A00–B99	Certain infectious and parasitic diseases	35 (4)	5 (2)
II	C00–D48	Neoplasm	167 (20)	65 (32)
III	D50–D89	Diseases of the blood and blood-forming organs and certain disorders involving the immune mechanism	5 (0.6)	1 (0.5)
IV	E00–E90	Endocrine, nutritional and metabolic diseases	10 (1)	1 (0.5)
VI	G00–G99	Diseases of the nervous system	8 (1)	1 (0.5)
IX	I00–I99	Diseases of the circulatory system	255 (30)	67 (33)
X	J00–J99	Diseases of the respiratory system	132 (15)	33 (16)
XI	K00–K93	Diseases of the digestive system	54 (6)	2 (1)
XII	L00–L99	Diseases of the skin and subcutaneous tissue	8 (1)	1 (0.5)
XIII	M00–M99	Diseases of the musculoskeletal system and connective tissue	131 (15)	10 (5)
XIV	N00–N99	Diseases of the genitourinary system	21 (2)	6 (3)
XX	V01–Y98	External causes of morbidity and mortality	16 (2)	3 (1)
Other chapters	–	–	5 (0.6)	1 (0.5)
Not coded	–	–	9 (1)	8 (4)

* Values are the number (%) of patients. Only those chapters of the International Statistical Classification of Diseases and Related Health Problems, Tenth Revision (ICD-10) that were used to classify >5 deaths are shown. Other causes of death included mental and behavioral disorders (2 patients in the anti-TNF cohort), disease of the ear and mastoid process (1 patient in the anti-TNF cohort), congenital malformations (1 patient in the anti-TNF cohort), injury or poisoning (1 patient in the nonbiologic DMARD cohort), and symptoms, signs, and abnormal clinical and laboratory findings (1 patient in the anti-TNF cohort). See Table 1 for other definitions.

**Figure 1:**
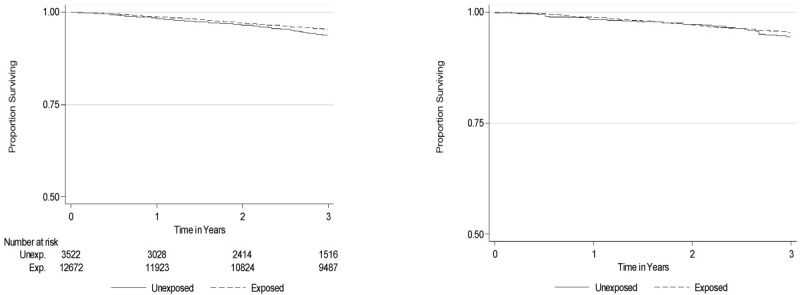
Kaplan-Meier survival curves showing all-cause mortality, both before adjustment (**left**) and after adjustment (**right**) for baseline differences, among patients receiving anti–tumor necrosis factor therapy (exposed) versus patients receiving nonbiologic DMARD therapy (unexposed).

All-cause mortality rates are presented in Table 3. Within the nonbiologic DMARD cohort, the unadjusted mortality rates differed based on whether or not complete baseline data on covariates were present, with a higher mortality rate among those patients with missing data. This effect was not observed in the anti-TNF cohort. After imputing data on missing covariates and adjusting for baseline differences, we found no overall difference in mortality rates between the anti-TNF cohort and the nonbiologic DMARD cohort (weighted HR 0.86 [95% CI 0.64–1.16]) (Figure 1). A similar pattern of mortality risk was found for each of the 3 leading causes of death within the cohort as follows: for circulatory disease, weighted HR 0.73 (95% CI 0.44–1.23), for neoplasm, weighted HR 0.65 (95% CI 0.39–1.09), and for respiratory disease, weighted HR 0.81 (95% CI 0.36–1.83). There was no evidence that the influence of treatment changed with the propensity score (*P* = 0.6) or that the assumption of proportional hazards was violated (*P* = 0.5). Truncating the IPTW at 20 did not affect the estimate of the HRs, nor did any of the following: excluding patients with weights >20, excluding untreated subjects with propensity scores lower than any treated subject, and excluding treated subjects with propensity scores higher than any untreated subject.

**3:** All-cause mortality rates in the anti-TNF and nonbiologic DMARD cohorts*

	Anti-TNF cohort			Nonbiologic DMARD cohort			
			
	No. of patients	Person-years	Mortality rate per 1,000 person-years (95% CI)	No. of patients	Person-years	Mortality rate per 1,000 person-years (95% CI)	HR
Patients with complete baseline data, unadjusted analysis	9,788	38,488	16.6 (15.4–17.9)	2,518	6,767	19.1 (16.1–22.6)	0.83 (0.68–1.00)
Patients with incomplete baseline data, unadjusted analysis	2,884	12,315	17.6 (15.4–20.1)	1,004	2,678	28.0 (22.3–35.1)	0.60 (0.46–0.79)
All patients, unadjusted analysis	12,672	50,803	16.8 (15.8–18.0)	3,522	9,445	21.6 (18.8–24.8)	0.74 (0.63–0.86)
All patients, weighted analysis	12,672	50,026	16.3 (14.9–17.8)	3,522	9,836	17.9 (13.5–23.6)	0.86 (0.64–1.16)

* 95% CI = 95% confidence interval; HR = hazard ratio (see Table 1 for other definitions).

## DISCUSSION

The results of this large national prospective cohort study have demonstrated that, when compared with standard nonbiologic DMARD therapy, anti-TNF therapy is not associated with either a significant increase or decrease in mortality. Furthermore, the individual causes of death did not differ substantially between these two groups, suggesting that, in the short term (mean 4 years), anti-TNF therapy does not seem to convey any additional risk or benefit on overall mortality risk when compared with standard nonbiologic DMARD therapy.

The findings in this study are in contrast to the findings of 2 previous studies on mortality and anti-TNF therapy: one from the Base de Datos de Productos Biológicos de la Sociedad Española de Reumatología in Spain and the other from the South Swedish Arthritis Treatment Group, both of which showed a substantial reduction in mortality among patients receiving anti-TNF therapy compared with that among patients receiving standard DMARD therapy (13,14). Unfortunately, the relatively small size of these studies (20 deaths in the Spanish study and 51 deaths in the Swedish study) prevented a detailed breakdown of the underlying causes of death. Furthermore, due to differences in study design, there was a limitation as to which confounders could be considered in the analyses.

The Spanish study was only able to adjust the full analysis by age and sex, after which a mortality ratio of 0.32 (95% CI 0.20–0.53) in anti-TNF–treated patients versus control patients was found. It is interesting to note that, in the Spanish study, the standardized mortality ratio in anti-TNF–treated patients, primarily recruited prior to 2002, was only 0.5. This is significantly lower than that which might be expected among a group of patients with severe RA requiring anti-TNF therapy, suggesting that a degree of channeling bias was present. The Swedish study included adjustment for a wide variety of disease-related features, including disease activity and disability, although adjustment for comorbidity was limited to COPD, diabetes, and cardiovascular disease. The authors of that study still found a reduction in overall mortality in anti-TNF–treated patients (HR 0.65 [95% CI 0.46–0.93]), although this reduction was only statistically significant among female patients in a further analysis stratified by sex.

One of the biggest challenges of these types of analyses is balancing the differences between patients receiving anti-TNF therapy and patients receiving nonbiologic DMARD therapy. By definition, patients with the most severe disease will go on to receive anti-TNF therapy. Similarly, patients who have a contraindication to anti-TNF therapy but have ongoing high disease activity may not receive treatment, and thus, the decision to treat or not to treat is not random but is based highly on a number of patient-related factors, many of which also influence the risk of death. This may explain why we observed a higher proportion of deaths from severe RA in the anti-TNF–treated group but a higher proportion of deaths from neoplasm, a relative contraindication for treatment (18), in the control group. Indeed, when confounding factors were not taken into consideration, the unadjusted mortality estimate in the current analysis also suggested lower death rates in the anti-TNF group (HR 0.74 [95% CI 0.63–0.86]), similar to the findings of previous studies (13,14).

In this study, we also observed a small proportion of patients who were originally enrolled in the DMARD cohort and then consented to further enrollment in the anti-TNF cohort when starting anti-TNF therapy. It is possible that these patients may have been healthier than those who remained in the DMARD cohort or those who were originally registered in the anti-TNF cohort, and thus, may have reduced the observed mortality risk as well, although the size of this cohort makes a significant effect on our results unlikely.

One option for balancing observed differences between treated and untreated subjects is to employ a propensity score. There are a number of ways of using the propensity score to adjust for baseline differences between treatment groups, which can be thought of as assessing the mean effect of treatment in different populations (22). The IPTW method used in the current study compares the expected outcome if all subjects were to switch to anti-TNF therapy with the expected outcome if all subjects were to continue on DMARDs. It is important to note that the comparison presented is between a plan to switch to anti-TNF treatment and a plan to continue with nonbiologic DMARD treatment, with the decision being made when the subject enters the study. Therefore, both arms of the analysis (both the anti-TNF cohort and the DMARD cohort) should ideally be as similar as possible at that specific point in time.

The strength of this study is its size and the vast collection of potential confounding information at baseline, as well as the prospective collection of followup data. We also had the benefit of a true internal comparison cohort of patients for whom the same detailed baseline information was collected. Because of the work of the NHS-IC, we were also assured of almost 100% followup of a patient's vital status. The cause of death was known in all but 17 patients. In a number of these missing cases, death occurred outside the UK, and no death certificate was available, although the death and date of death were confirmed by a rheumatologist, thus allowing inclusion in our all-cause mortality analysis.

A potential limitation of the current study was the effect of missing data at baseline. Although every effort was made to collect comprehensive data on all patients, in many cases, certain elements of the baseline covariates were missing. It was interesting to observe that the mortality rate was markedly higher among comparison subjects with incomplete data (28 per 1,000 person-years) than among comparison subjects with complete data (19 per 1,000 person-years). The unadjusted HR was 0.83 among subjects with complete data, compared with 0.74 in all subjects, an overestimate of 12%. The reasons for this discrepancy are not entirely clear. The most frequent missing data item in the nonbiologic DMARD group was the HAQ score. It may be that patients with multiple comorbidities or an element of frailty were not able to complete these forms, thus introducing the potential for bias into our data. However, although there were missing data items, we were not missing the patient information required to impute missing data. Indeed, <2% of information was missing with regard to calculation of HRs in the weighted analysis. This justifies the use of multiple imputation to obtain an unbiased estimate in our analysis.

One risk of studying all-cause mortality is that there may be individual trends within specific causes of death that may be overlooked. The 3 leading causes of death in the current study were circulatory disease, neoplasm, and respiratory disease. We have previously shown that the overall risk of early myocardial infarction was similar between our anti-TNF and nonbiologic DMARD groups, although patients whose RA had responded to the initial anti-TNF agent at 6 months had lower rates of myocardial infarction (11). In the current study, although we saw a trend toward a reduction in death from circulatory disease, this did not reach statistical significance. Similarly, we saw a reduced point risk estimate for death due to neoplasm in the anti-TNF cohort, although again this did not reach statistical significance. It is possible that, with longer followup, a different pattern of risk from that observed in this study may emerge. In addition, since many of these diagnoses will not be fatal over a short period, the data cannot be used to extrapolate information on the differences in overall incidence of cardiac disease and neoplasm in the 2 cohorts.

A remaining challenge in the analysis of observational data is an understanding of the role that baseline comorbidities have in the risk of cause-specific death. In the current study, we were able to adjust for a number of comorbidities present at baseline; however, we were not able to adjust for the severity of these comorbidities. It is possible that there remains a channeling bias of healthier patients toward anti-TNF therapies, which, in part, may explain the trends observed in some of our results. However, a suggestion that disease control can also have beneficial effects on long-term outcomes suggests that this is not the only explanation for these trends.

We did not include a specific analysis of infection as an underlying cause of death. First, infection is not a single category within the ICD but instead crosses many ICD chapters. In addition, this analysis included the underlying cause of death and not the immediate cause of death, which is where infection may be more likely to be categorized (e.g., death from pneumonia following cerebrovascular accident). However, previous studies have shown that serious infections (those resulting in hospitalization and/or death) are increased in patients receiving anti-TNF therapy, particularly in the early months of treatment. Whether this is related to a total increase in death from infection over time is less clear.

In conclusion, this analysis of all-cause and cause-specific mortality in a large UK cohort of patients with severe RA has demonstrated that treatment with anti-TNF therapies over a mean of 4 years is not associated with an increased risk of death, as compared with treatment with standard DMARD therapy alone. Future analysis will confirm if this trend in mortality, including the underlying causes of death observed in both cohorts, is maintained over longer periods of followup.

## APPENDIX A: BSRBR CONTROL CENTRE CONSORTIUM

The BSRBR Control Centre Consortium consists of the following institutions (all in the UK): Antrim Area Hospital, Antrim (Dr. Nicola Maiden), Cannock Chase Hospital, Cannock Chase (Dr. Tom Price), Christchurch Hospital, Christchurch (Dr. Neil Hopkinson), Derbyshire Royal Infirmary, Derby (Dr. Sheila O'Reilly), Dewsbury and District Hospital, Dewsbury (Dr. Lesley Hordon), Freeman Hospital, Newcastle-upon-Tyne (Dr. Ian Griffiths), Gartnavel General Hospital, Glasgow (Dr. Duncan Porter), Glasgow Royal Infirmary, Glasgow (Professor Hilary Capell), Haywood Hospital, Stoke-on-Trent (Dr. Andy Hassell), Hope Hospital, Salford (Dr. Romela Benitha), King's College Hospital, London (Dr. Ernest Choy), Kings Mill Centre, Sutton-In-Ashfield (Dr. David Walsh), Leeds General Infirmary, Leeds (Professor Paul Emery), Macclesfield District General Hospital, Macclesfield (Dr. Susan Knight), Manchester Royal Infirmary, Manchester (Dr. Ian Bruce), Musgrave Park Hospital, Belfast (Dr. Allister Taggart), Norfolk and Norwich University Hospital, Norwich (Professor David Scott), Poole General Hospital, Poole (Dr. Paul Thompson), Queen Alexandra Hospital, Portsmouth (Dr. Fiona McCrae), Royal Glamorgan Hospital, Glamorgan (Dr. Rhian Goodfellow), Russells Hall Hospital, Dudley (Professor George Kitas), Selly Oak Hospital, Selly Oak (Dr Ronald Jubb), St. Helens Hospital, St. Helens (Dr. Rikki Abernethy), Weston General Hospital, Weston-super-Mare (Dr. Shane Clarke/Dr. Sandra Green), Withington Hospital, Manchester (Dr. Paul Sanders), Withybush General Hospital, Haverfordwest (Dr. Amanda Coulson), North Manchester General Hospital, Manchester (Dr. Bev Harrison), Royal Lancaster Infirmary, Lancaster (Dr. Marwan Bukhari), and The Royal Oldham Hospital, Oldham (Dr. Peter Klimiuk).
